# AMPA receptors modulate enhanced dopamine neuronal activity induced by the combined administration of venlafaxine and brexpiprazole

**DOI:** 10.1038/s41386-024-01958-4

**Published:** 2024-08-15

**Authors:** Stephen Daniels, Mostafa El Mansari, Pierre Blier

**Affiliations:** University of Ottawa Institute of Mental Health Research, 1145 Carling Avenue, K1Z 7K4, Ottawa, Canada

**Keywords:** Preclinical research, Depression

## Abstract

Addition of dopamine (DA)/serotonin (5-HT) partial agonists to 5-HT/norepinephrine (NE) reuptake inhibitors are commonly used to enhance the antidepressant response. The simultaneous inhibition of 5-HT and NE transporters with venlafaxine and its combination of brexpiprazole, which blocks the α_2_-adrenergic autoreceptor on NE terminals, could constitute a superior strategy. Anesthetized rats received venlafaxine and brexpiprazole for 2 and 14 days, then the firing activity of dorsal raphe nucleus 5-HT, locus coeruleus NE, and ventral tegmental area DA neurons were assessed. Net 5-HT and NE neurotransmissions were evaluated by assessing the tonic activation of 5-HT_1A_, and α_1_- and α_2_-adrenergic receptors in the hippocampus. The combination of brexpiprazole with venlafaxine resulted in normalized 5-HT and NE neuron activity, which occurred earlier than that with venlafaxine alone. A significant enhancement of the tonic activation of 5-HT_1A_ receptors and α_2_-adrenoceptors in the hippocampus was observed following administration of the combination for 14 days. The combination more than doubled the number of DA neurons per electrode descent, after both 2 and 14 days, while this increase was observed only after 14 days of venlafaxine administration. This increase in population activity was prevented by NBQX, an AMPA receptor antagonist. In conclusion, early during administration, the combination of venlafaxine with brexpiprazole normalized firing activity of 5-HT and NE neurons, and increased the population activity of DA neurons through AMPA receptors. In the hippocampus, there was an overall increase in both 5-HT and NE transmissions. These results imply that this strategy could be a rapid-acting approach to treat depression.

## Introduction

Despite substantial progress in the area of depression research, current treatments for major depressive disorder (MDD) remain far from optimal. Only a third of patients with MDD achieve remission following treatment with initial first-line medications, like selective serotonin (5-HT) reuptake inhibitors (SSRIs) or 5-HT and norepinephrine (NE) reuptake inhibitors (SNRIs). Adjunctive strategies with different mechanisms of action than those of SSRIs and/or SNRIs are often used following an inadequate response in patients with MDD. One such approach is the addition, at low doses, of medications initially devised to treat schizophrenia, because of their high affinity for several monoaminergic receptors [1, 2].

While SSRIs administered either acutely or repeatedly were shown to increase 5-HT transmission [3], they decrease the firing rate and burst activity of the dopamine (DA) and NE neurons via the increased activation of 5-HT_2C_ and 5-HT_2A_ receptors (5-HT_2C_Rs and 5-HT_2A_Rs), respectively [4–6]. By antagonizing these receptors, low doses of medications such as olanzapine, risperidone, and DA/5-HT partial agonists counterbalance the inhibitory actions of SSRIs on catecholamine neurons [5, 7–9]. The ensuing reversal of dampened DA and NE tone could thus be of crucial importance in the treatment of MDD. SNRIs, on the other hand, by increasing NE synaptic levels throughout the brain [10, 11] also induce a sustained decrease in NE neurons firing [12].

Brexpiprazole is efficacious as an add-on in depressed patients with an inadequate response [13]. It possesses common properties with other DA/5-HT modulators used as adjunctive strategies. It acts as a D_2_ receptor partial agonist, like aripiprazole [1]. Both agents are also 5-HT_1A_R agonists, similar to buspirone and gepirone—agents that are shown to be effective in depression when used as a monotherapy and in combination [14, 15]. They also have a very high affinity for 5-HT_2A_R, the blockade of which reverses the inhibitory action of SSRI on NE neuronal firing [5, 7, 8]. Brexpiprazole, however, stands out because of its antagonistic action of α_2C_-adrenoceptors. Unlike the 5-HT_2A_R antagonist MDL100907, brexpiprazole and the α_2_-adrenergic idazoxan both increase the firing activity of NE neurons when administered alone [16, 17].

Brexpiprazole is an effective adjunctive strategy for inadequate response in patients with MDD when the 5-HTT is inhibited. It could putatively be a superior strategy if used at doses that significantly engage the NE transporter (NET) as well (venlafaxine 225 mg or duloxetine 120 mg per day) [18–21]. This would result from the synergy between the NE transporter and the terminal α_2_-adrenergic autoreceptor as was documented in rats. Indeed, NET inhibition increases extracellular NE by about 300% whereas α_2_-adrenoceptor inhibition enhances NE concentration by about 100%, yet when combined the yield is a 500–600% augmented level [22, 23].

In the present study, the electrophysiological effects of the simultaneous combination of venlafaxine and brexpiprazole (termed the combination herein) on firing activity of 5-HT, NE, and DA neurons were first examined. Then the overall net effect of the combination was also determined in a projection area, namely the hippocampus.

## Methods and materials

### Animals

Male Sprague-Dawley rats (Charles River, St-Constant, QC) weighing 250–350 g were used at the time of the recordings. Rats were housed in a controlled environment with a (12:12) light-dark cycle and ad libitum access to food and water. Upon arrival at the facility, rats were allowed at least 5 days of acclimation to laboratory conditions. All experiments were conducted by the local Animal Care Committee (University of Ottawa, Ottawa, ON, Canada) and the Canadian Council on Animal Care.

### Drug treatments

Although routes of administration for venlafaxine and brexpiprazole were different, previous data showed that they adequately engaged their targets. The current study used doses of venlafaxine and brexpiprazole that have been shown to act on intended receptors and transporters, probably mimicking its effect in patients with depression. For venlafaxine, the regimen used in the current study blocked in a sustained manner 5-HT and NE transporters, as in the clinic. For brexpiprazole, a dose of 1 mg/kg exerted a partial agonism at D_2_ and 5-HT_1A_ receptors, antagonism at 5-HT_2A_ receptors, and an antagonistic action of α_2C_-adrenoceptors. In addition, there is no pharmacokinetic interaction between venlafaxine and brexpiprazole, as both being substrates of cytochrome P450 2D6. These two medications are both metabolized by this iso-enzyme, but they do not inhibit its activity [12, 16].

Venlafaxine was administered for 2 or 14 days using Alzet minipumps implanted subcutaneously under isoflurane anesthesia. The regimen (40 mg/kg/day) was based on the results of our prior experiments in rats, as it was shown to block NE and 5-HT transporters [12, 24].

Brexpiprazole was subcutaneously injected daily at a dose of 1 mg/kg/day as previously reported [16]. The last injection was given 30 min before electrophysiological recordings. All experiments were conducted with drugs on board during electrophysiological recordings. Venlafaxine and brexpiprazole were dissolved, respectively, in saline and 2-hydroxy-beta-cyclodextrin (20%), which were used as a vehicle. Control groups included the implantation of an osmotic minipump-containing saline and were given 2-hydroxy-beta-cyclodextrin injections daily.

### In vivo electrophysiological recordings

The electrophysiological recordings were obtained under chloral hydrate anesthesia (400 mg/kg, i.p.) with supplemental doses given (100 mg/kg) to prevent any nociception with the head of the rats fixed in a stereotaxic frame. A burr hole was drilled through the skull to record, respectively, ventral tegmental area (VTA) DA, dorsal raphe nucleus (DRN) 5-HT, locus coeruleus (LC) NE, and CA3 pyramidal neurons of the hippocampus.

### Electrophysiological recording of VTA DA neurons

The recording of DA neurons was obtained with a single-barreled glass micropipette lowered at 3.0–3.6 mm anterior to lambda and 0.6–1.0 mm lateral to the midline suture. These neurons were encountered at a depth of 6.0 to 8.5 mm from the surface of the brain. The presumptive DA neurons were identified by the established electrophysiological criteria: regular or irregular single spiking pattern that may include burst firing with a rate between 2 and 10 Hz; biphasic or triphasic waveforms, with a notch, and a duration >1.1 ms from start to trough of the waveform; long-duration action potentials (2.5–4 ms), and low-pitch sound when monitored by an audio amplifier [25]. The number of spontaneously active DA neurons found per track was determined by recording multiple tracks in a grid of 6–9 tracks per rat.

### Electrophysiological recording of DRN 5-HT neurons

To record 5-HT neurons, a single-barreled micropipette was positioned 0.9–1.2 mm anterior to lambda on the midline and lowered into the DRN. The putative 5-HT neurons were encountered over 1 mm immediately below the ventral border of the Sylvius aqueduct and identified by their slow (0.5–2.5 Hz), regular firing rate, and long duration (~2 ms) positive action potential [26].

### Electrophysiological recording of LC NE neurons

NE neurons were recorded with a single-barreled glass micropipette positioned at 1.1–1.2 mm posterior to lambda and 0.9–1.3 mm lateral to the midline suture. These neurons were encountered at a depth of 4.5 to 6.0 mm from the surface of the brain. The NE neurons were identified by their regular firing rate (0.5–5 Hz), an action potential of long duration (~2 ms), and a characteristic volley of spikes discharge followed by a quiescent period in response to a nociceptive pinch of the contralateral hind paw [27].

### Burst analysis

The firing patterns of the monoaminergic neurons were analyzed by interspike interval (ISI) burst analysis. The onset of a burst was signified by the occurrence of 2 spikes with ISI < 0.08 s for NE and DA, and ISI < 0.01 s for 5-HT. The termination of a burst was defined as an ISI > 0.16 s for NE and DA, and ISI > 0.01 s for 5-HT [28, 29]. Burstidator software (https://github.com/nno/burstiDAtor) was used for bursting analysis.

### Extracellular recording and microiontophoresis of dorsal hippocampus CA3 pyramidal neurons

The multi-barrel electrode was lowered into the CA3 region of the hippocampus using the following coordinates: 4–4.2 mm anterior to lambda and 4.2 mm lateral. Pyramidal neurons were encountered at a depth of 4.0 ± 0.5 mm below the surface of the brain. They were activated with a small current (in nanoAmpere [nA]) of quisqualic acid within their physiological firing range (10–15 Hz) because these neurons do not discharge spontaneously in chloral hydrate anesthetized rats. The CA3 pyramidal neurons were identified by their large amplitude (0.5–1.2 mV) and long-duration simple action potentials, alternating with complex spike discharges. Extracellular recording of CA3 pyramidal neurons and iontophoretic applications were performed with five-barreled glass micropipettes. The impedance of these electrodes ranges from 2 to 4 MΩ. The central barrel used for the unitary recording was filled with a 2 M NaCl solution, and the four side barrels were filled with the following solutions: 5-HT creatinine sulfate (20 mM in 200 mM NaCl, pH 4), (±)-NE bitartrate (20 mM in 200 mM NaCl, pH 4), quisqualic acid (1.5 mM in 200 mM NaCl, pH 8), and a 2 M NaCl solution used for automatic current balancing.

### In vivo determination of NE and 5-HT uptake

To confirm the relative degree to which venlafaxine blocks NET and 5-HT transporter (5-HTT), the RT50 values were determined after the microiontophoretic application of NE and 5-HT on CA3 pyramidal neurons. The RT50 values correspond to the time in seconds (s) elapsed from the cessation of microiontophoretic application of NE/5-HT to 50% recovery of the initial firing rate [30].

### In vivo determination of the sensitivity of 5-HT_1A_Rs and α-adrenoceptors

Pyramidal neurons sensitivity to the microiontophoretic application of 5-HT/NE was quantified by means of the IT50 index (nC) i.e., the current (nA) multiplied by the time (s) required to obtain 50% inhibition of spontaneous firing rate of pyramidal neurons [31].

### Determination of the tonic activation of 5-HT_1A_, α_1_- and α_2_-adrenergic receptors in the hippocampus

To assess the degree of activation of the 5-HT_1A_, α_1-_ and α_2_-adrenergic receptors exerting an inhibitory influence on the firing activity of CA3 pyramidal neurons, WAY 100,635 (25–100 µg/kg), prazosin (0.1 mg/kg) and idazoxan (1 mg/kg) were intravenously administered to disinhibit these neurons, respectively [32, 33].

### Determination of the effect of electrical stimulation of the afferent 5-HT pathway on the activity of hippocampal pyramidal neurons

To electrically stimulate the ascending 5-HT pathway a bipolar electrode (NE-100, David Kopf, Tujunga, CA, USA) was implanted 1 mm anterior to lambda on the midline with a 10° backward angle in the ventromedial tegmentum and 8.0 ± 0.2 mm below the surface of the brain. Two hundred square pulses with a duration of 0.5 ms were delivered by a stimulator (S48, Grass Instruments, West Warwick, RI, USA), at an intensity of 300 µA and a frequency of 1 and 5 Hz. The stimulation of the 5-HT pathway induces a brief suppressant period due to the release of 5-HT into the synapse [34]. Peristimulus time histograms (PSTH) of CA3 pyramidal neurons were generated to determine duration of suppression (DOS in ms) of firing. DOS is defined as the time interval initiated by a 50% reduction in the number of events per bin (width of 2 ms) from the mean prestimulation rate of firing to the time it returned to 90% of that same prestimulation value. The effect of stimulation of the ascending 5-HT pathway (using 1 and 5 Hz) was assessed on the same neuron, to determine the function of terminal 5-HT_1B_ autoreceptors [35].

### Data analysis

Data are presented as mean values ± S.E.M. Data points for each rat were averaged and used for statistical comparisons. As indicated in the results section, statistical comparisons between rats that received vehicle and those receiving drug treatment were carried out using a two-tailed *t*-test, the non-parametric Mann–Whitney or One-Way analysis of variance (ANOVA) with a post-hoc analysis when appropriate. For the tonic activation and stimulation data, statistical comparisons were carried out using a two-way analysis of variance with or without repeated measures (treatment as the main factor) followed by post hoc analysis for all pairwise multiple comparisons. Statistical comparisons were done using the software SigmaPlot 12.5 (Systat Software Inc, San Jose, CA, USA).

### Drugs

Venlafaxine was purchased from LKT Laboratories Inc. (Saint Paul, MN, USA). Brexpiprazole was purchased from BOC Sciences (Shirley, NY, USA). Chloral hydrate, WAY 100635, 5-HT creatinine sulfate, NE bitartrate, idazoxan, and prazosin were purchased from Sigma (Oakville, ON, Canada). Quisqualic acid was purchased from Tocris (Ellisville, MO, USA). NBQX was purchased from Cayman Chemical (Cederlane, Burlington, ON, Canada)

## Results

### Effect of 2- and 14-day administration of venlafaxine alone and in combination with brexpiprazole on the activity of VTA DA neurons

**Fig. 1:**
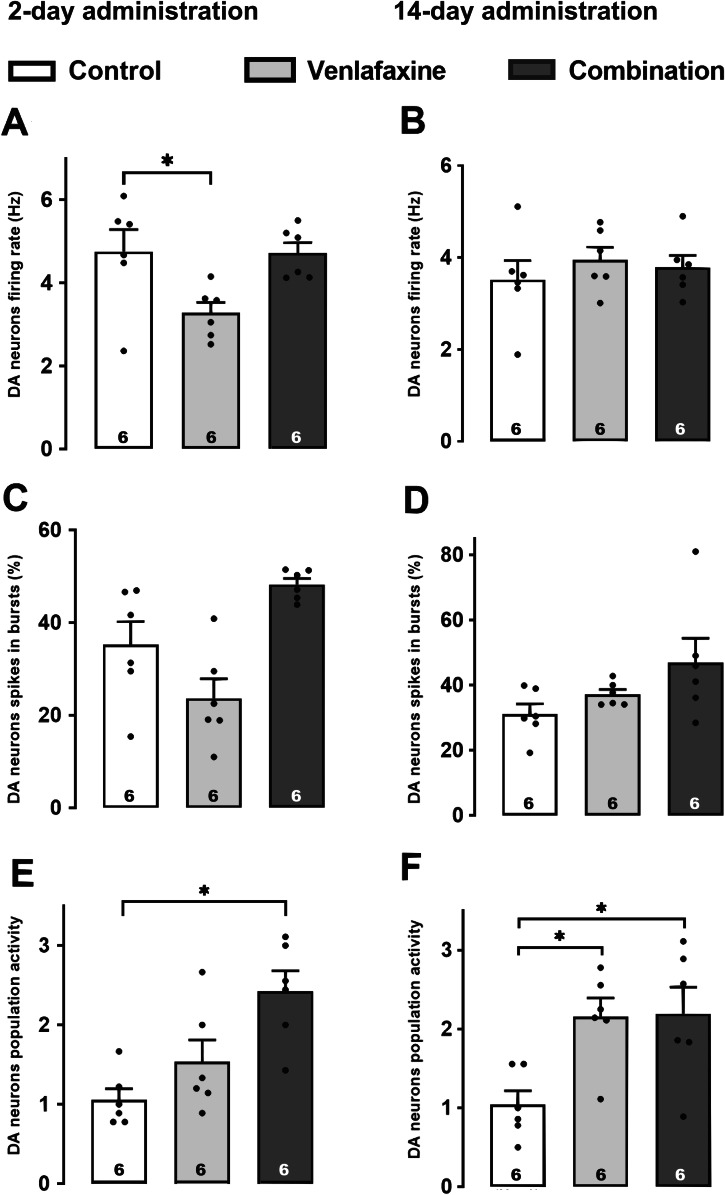
Effect of short-term and sustained administration of the combination of venlafaxine and brexpiprazole on VTA DA neurons. Histograms show firing activity (**A**, **B**), percentage of spikes firing in bursts (**C**, **D**), and population activity (**E**, **F**) in rats that received the vehicle (2 days: number of neurons = 57 versus 14 days: number of neurons = 54), venlafaxine (2 days: number of neurons = 76 versus 14 days: number of neurons = 110), and combination (2 days: number of neurons = 128 versus 14 days: number of neurons = 104). The histograms show data as mean values ± SEM. Each dot represents data from one rat. Number of rats is indicated at the bottom of the histogram. Statistical significance is indicated where it applies, *P < 0.05.

Venlafaxine administered for 2 days decreased significantly by 30% firing activity of DA neurons when compared to the vehicle group. However, when combined with brexpiprazole for 2 days, neuronal firing activity of these neurons was at to control level (Fig. 1A; One-way ANOVA followed by Bonferroni method, *F*[2,15] = 5.2, *p* = 0.02). After 14 days of administration, however, there was no decrease in firing with venlafaxine nor with venlafaxine plus brexpiprazole (Fig. 1B; One-way ANOVA followed by Bonferroni method, *F*[2,15] = 0.5, *p* = 0.6).

Although venlafaxine alone did decrease the percentage of spikes in burst by 33%, after 2 days of administration, this was not significant. Its combination with brexpiprazole resulted in an important, but not significant increase following 2- (37%) and 14-day administration (47%; Fig. 1C, D).

The population activity of DA neurons was not significantly altered by venlafaxine administered for 2 days, but caused a significant increase after 14 days of administration, compared to the control group (Fig. 1E, F; One-way ANOVA followed by Bonferroni test, *F*[2,15] = 6.4, *p* < 0.01). A similar enhancement was obtained after only 2 days of administration with the combination (One-way ANOVA followed by Bonferroni test, *F*[2,15] = 9, *p* = 0.003). This increase was maintained after 14 days in comparison with the control group (One-way ANOVA followed by Bonferroni test, *F*[2,15] = 6.4, *p* = 0.01).

### Effect of the α-amino-3-hydroxy-5-methyl-4-isoxazolepropionic acid (AMPA) receptor antagonist 2,3-dioxo-6-nitro-7-sulfamoyl-benzo[f]quinoxaline (NBQX) on short-term administration of venlafaxine in combination with brexpiprazole-induced increase in population activity of DA neurons

Glutamate was previously reported to be involved in modulating population activity of DA neurons [36, 37]. In the current study, we tested whether AMPAR is involved in the combination-induced enhancement of DA neuron population activity, by blocking these receptors by NBQX [38].

**Fig. 2:**
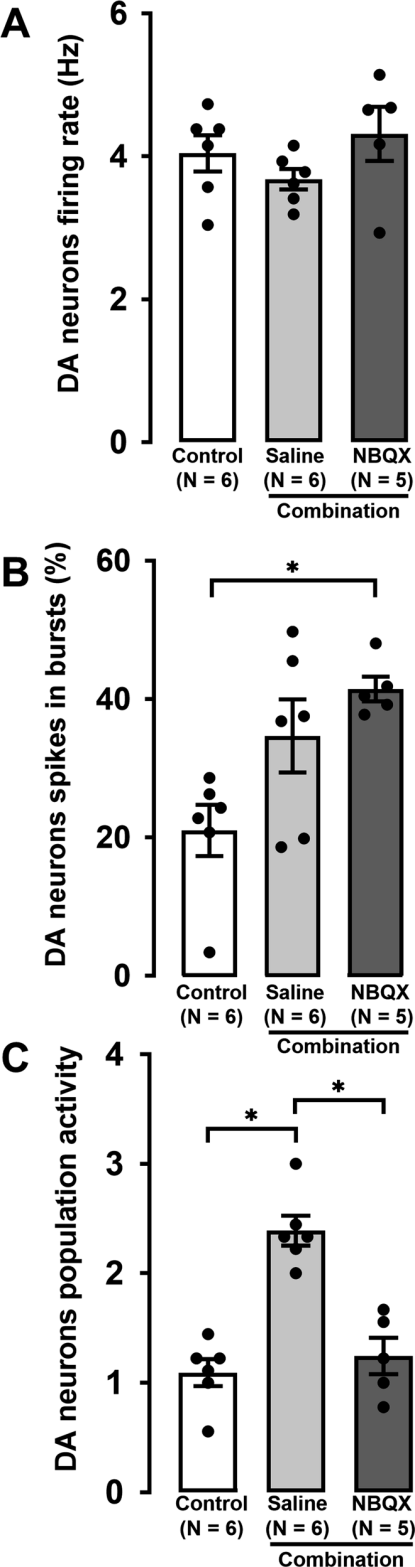
Effect of pretreatment with NBQX (10 mg/kg; i.p.) administered 30 min before VTA DA neuron recordings in rats administered the combination of venlafaxine and brexpiprazole for 2 days. Histograms show firing rate (**A**), percentage of spikes firing in burst mode (**B**), and population activity (**C**) in the control group (number of neurons = 56) and combination group that was pretreated with saline (number of neurons = 113) or NBQX (number of neurons = 60). The histograms show data as mean values ± SEM. Each dot represents average of all recorded neurons from one rat. Number of rats is indicated at the bottom of the histogram. Statistical significance is indicated where it applies, *P < 0.05.

Two groups of rats that received combination administration were randomly given saline or NBQX, 30 min before neuronal recordings and were compared to a vehicle group. In the three groups, the firing rate of DA neurons was not significantly different (Fig. 2A; One-way ANOVA *F*[2,14] = 1.4, *p* = 0.3). After 2-day administration, the combination significantly increased the percentage of spikes in burst in the presence of NBQX compared to the control group (Fig. 2B; One-way ANOVA *F*[2,14] = 6.5, *p* = 0.01). However, the combination-induced increase in DA neuron population activity was blocked in rats that received NBQX 30 min before the recording (Fig. 2C; *F*[2,14] = 23, *p* < 0.001).

### Effect of 2- and a 14-day administration of venlafaxine in combination with brexpiprazole on the activity of DRN 5-HT neurons

**Fig. 3:**
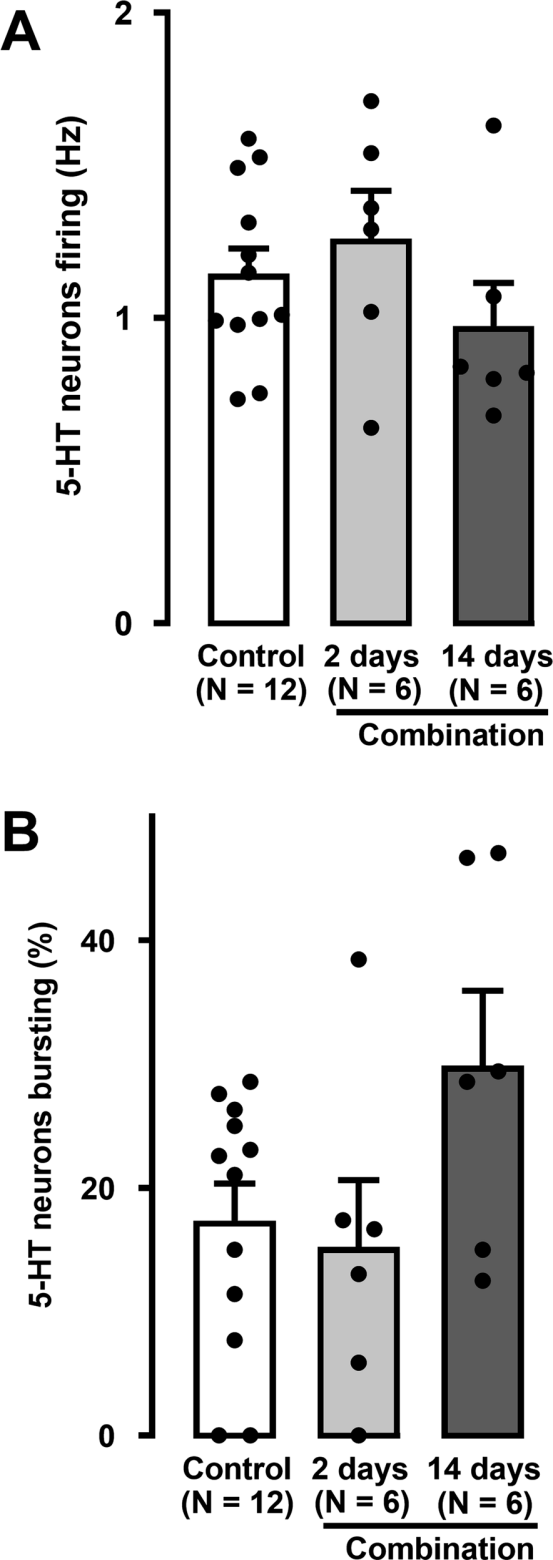
Effect of short-term and sustained administration of the combination of venlafaxine and brexpiprazole on DRN 5-HT neuronal activity. Histograms show firing activity (**A**) and the percentage of neurons with burst activity (**B**) in rats administered the combination for 2 (number of neurons = 107) or 14 days (number of neurons = 106) compared to the vehicle-administered control group (number of neurons = 254). The histograms show data as mean values ± SEM. Each dot represents average of all recorded neurons from one rat. Statistical significance is indicated where it applies.

There was no statistically significant difference in the firing activity of 5-HT neurons in the 2- and 14-day vehicle groups, hence the data from these two groups were combined. Following the addition of brexpiprazole to venlafaxine for 2 and 14 days, the mean firing rate of 5-HT neurons was similar to that found in the control group (Fig. 3A; One-way ANOVA, *F*[2,21] = 1.2, *p* = 0.3).

Furthermore, there were no significant differences in the percentage of 5-HT neurons firing in bursts in combination-administered rats for 2 and 14 days, when compared to vehicle-treated rats (Fig. 3B; One-way ANOVA, *F*[2,21] = 2.7, *p* = 0.09).

### Effect of sustained administration of the combination on the overall 5-HT transmission in CA3 region of the hippocampus

#### Sensitivity of 5-HT_1A_R

To determine the sensitivity of the 5-HT_1A_R located on CA3 pyramidal neurons, the IT50 index was calculated (the current to eject 5-HT [20 nA] multiplied by the time (s) required to obtain 50% inhibition of spontaneous firing rate of pyramidal neurons). The IT50 was not significantly different in combination group when compared to control rats (Table 1A; Mann–Whitney *U* = 6, *p* = 0.07).

**Table 1:** Effect of the combination of venlafaxine and brexpiprazole on the sensitivity (IT50) of the 5-HT_1A_R and the α_2_-adrenoreceptor and activity of 5-HT and NE transporter (RT50) in response to iontophoretic ejection of 5-HT (A) and NE (B), in the CA3 region of the hippocampus. Statistical significance is indicated where it applies. *P < 0.05.

			IT50		RT50	
			Mean	Rats	Mean	Rats
(A) 5-HT	14 days	Control	51 ± 7	6	34 ± 7	10
		Combination	33 ± 13	6	68 ± 13*	12
(B) NE	2 days	Control	56 ± 12	6	30 ± 11	11
		Combination	51 ± 7	7	83 ± 12*	6
	14 days	Control	101 ± 37	6	33 ± 9	12
		Combination	140 ± 31	6	26 ± 12	9

#### Activity of 5-HT transporters

Since the prolonged venlafaxine administration was previously shown to block 5-HTT and NET, we assessed the RT50 to ascertain that the effect of venlafaxine was present when this drug was combined with brexpiprazole.

In rats administered the combination for 14 days, the RT50 was significantly increased by iontophoretic ejections of 20 nA of 5-HT compared to control (Table 1A; Two-tailed *t*-test *t* [20] = –2.2, *p* = 0.04).

#### Tonic activation of 5-HT_1A_R

**Fig. 4:**
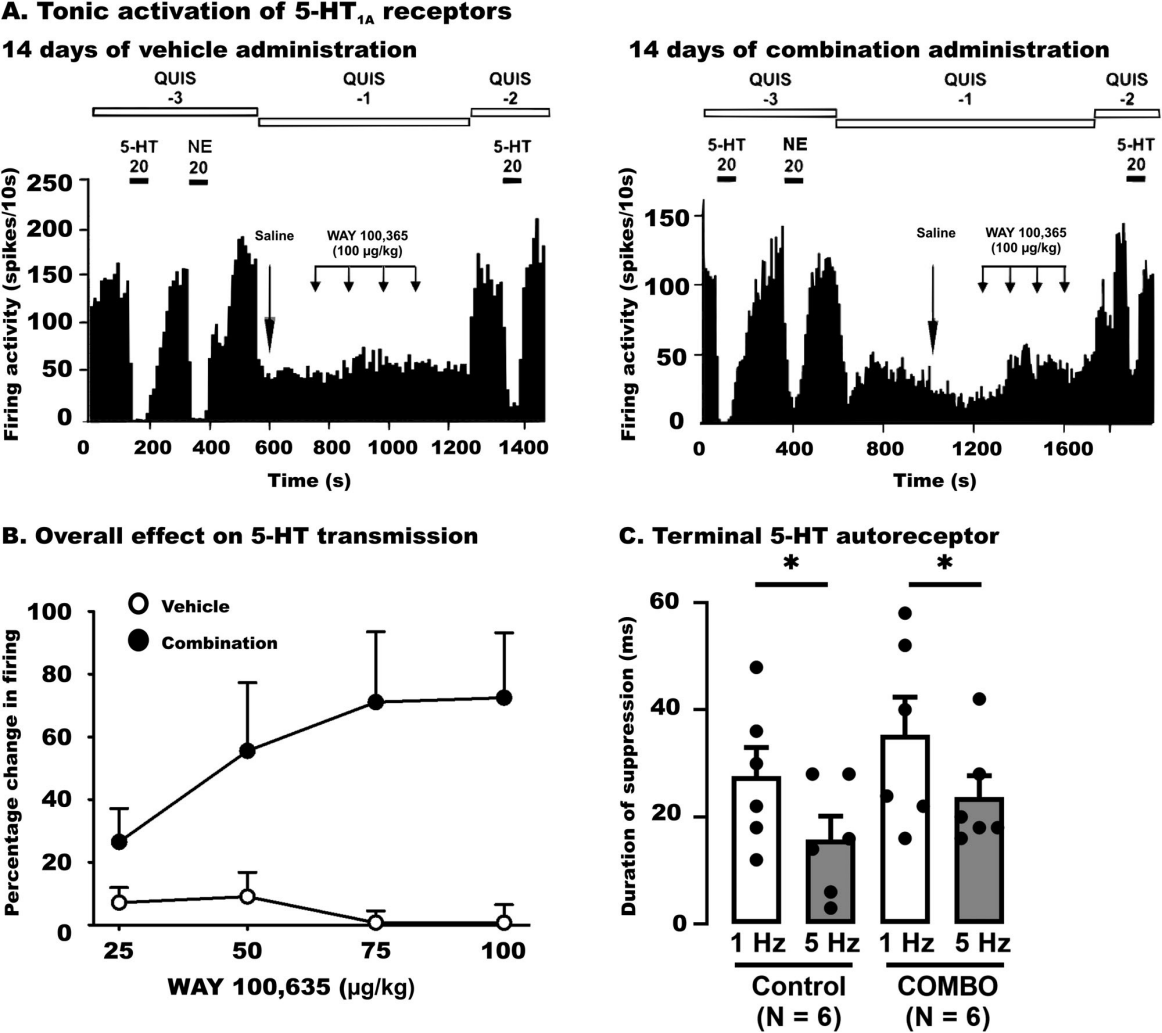
Effect of sustained administration of the combination of venlafaxine and brexpiprazole on the tonic activation of 5-HT_1A_ receptor in CA3 hippocampal pyramidal neurons. Illustrative traces (**A**) of the effect of cumulative WAY 100,635 (25–100 µg/kg) administration on the firing activity of a CA3 pyramidal neuron in a vehicle-administered rat, and a 14-day combination-administered rat. Overall change (%, **B**) in the firing activity of pyramidal neurons after the administration of WAY100635 in vehicle rats (*N* = 6) and those administered with combination for 14 days (*N* = 6). Only one neuron was tested in each rat. Data are presented as mean values ± S.E.M; Statistical significance is indicated where it applies. **C** Comparison of the duration of suppression produced by stimulation of the 5-HT afferent fiber bundle on CA3 pyramidal neurons in the vehicle- and 14-day combination administered rats. The histograms show data as mean values ± SEM. Each dot represents average of all recorded neurons from one rat. Statistical significance is indicated where it applies, *P < 0.05.

As illustrated in Fig. 4, for the tonic activation of 5-HT_1A_ receptors, two-way repeated measures ANOVA indicated a significant effect of treatment (*F*[1,30] = 8, *p* < 0.02), and dose (*F*[3,30] = 2, *p* = 0.2) but no interaction between group and dose (*F*[3,30] = 3, *p* = 0.052). When compared to the control group, 14-day administration of the combination, resulted in a significant increase in the percentage change in firing activity of CA3 pyramidal neurons following administration of WAY 100,635, although independently of the dose (Fig. 4A, B).

#### Responsiveness of the terminal 5-HT autoreceptor

As illustrated in Fig. 4C, using a two-way repeated measures ANOVA analysis, there was no significant interaction between the degree of stimulation and treatment (*F*[1,10] = 0.0004; *p* = 0.9). There was no significant difference in the duration of suppression between the group of rats that received combination compared to the vehicle (*F*[1,10] = 1.2; *p* = 0.3). This analysis elicited, however, a statistically significant effect of frequency of stimulation (*F*[1,10] = 22; *p* < 0.001), for which there was a significant difference between the duration of suppression induced by 1 Hz versus 5-Hz in vehicle and combination group.

### Effect of 2- and 14-day administration of venlafaxine in combination with brexpiprazole on the activity of LC NE neurons

There was no statistically significant difference in the firing activity of NE neurons in the 2- and 14-day control groups, consequently, the data for these groups were combined.

**Fig. 5:**
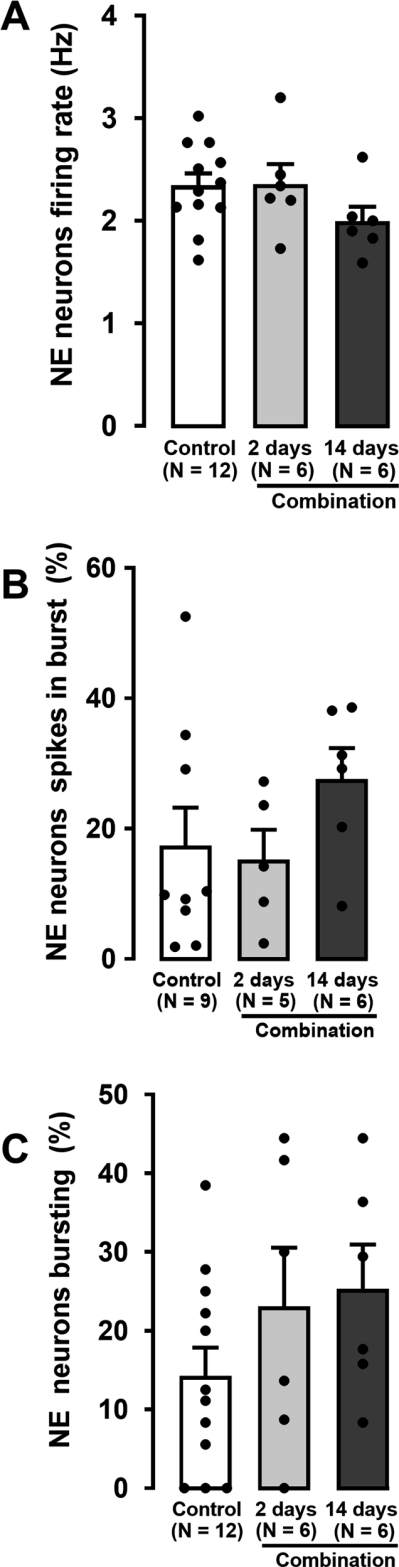
Effect of short-term and sustained administration of the combination of venlafaxine and brexpiprazole on LC NE neuronal activity. Histograms show firing activity (**A**), percentage spikes occurring in bursts (**B**), and percentage of neurons with burst activity (**C**) in rats administered the combination for 2 (number of neurons = 87) or 14 days (number of neurons = 94) compared to the vehicle-administered control group (the number of neurons = 171). The histograms show data as mean values ± SEM. Each dot represents average of all recorded neurons from one rat. Statistical significance is indicated where it applies.

Both after 2- and 14-day addition of brexpiprazole to venlafaxine, the firing activity of NE neurons was not significantly different from that found in rats receiving vehicle (Fig. 5A; One-way ANOVA, *F*[2,21] = 1.6, *p* = 0.2).

Percentage of spikes in burst firing of NE neurons was not statistically different between combination and control groups (Fig. 5B; One-way ANOVA, *F*[2,17] = 1.3, *p* = 0.3).

Similarly, there was no significant difference in the percentage of NE neurons with burst activity in rats administered the combination compared to control rats for either 2 or 14 days (Fig. 5C; control: 14 ± 4%; combination 2-day: 23 ± 8%; combination 14-day: 25 ± 6%; One-way ANOVA, *F*[2,21] = 1.5, *p* = 0.3).

### Effect of sustained administration of the combination on the overall NE transmission in CA3 region of the hippocampus

#### Sensitivity of α_2_-adrenoceptors

The responsiveness of the α_2_-adrenoceptors on CA3 pyramidal neurons was assessed by ejecting NE at a current of 20 nA. It showed that the mean IT50 for combination group was not significantly different from controls (Table 1B; Two-tailed *t*-test, *t* [10] = –39, *p* = 0.4).

#### Activity of NE transporters

Administration of combination for 14 days did not significantly alter the RT50 in response to the iontophoretic ejection of NE after 14 days of administration (Table 1B; Mann–Whitney *U* = 41, *p* = 0.4). To rule out that this lack of effect is not due to inadequacy of the venlafaxine itself, RT50 was determined in rats that received only venlafaxine. When administered alone for 2 days, venlafaxine more than doubled the RT50 compared to the control group (Table 1B; Mann–Whitney *U* = 78, *p* = 0.02).

#### Tonic activation of α-adrenoceptors

**Fig. 6:**
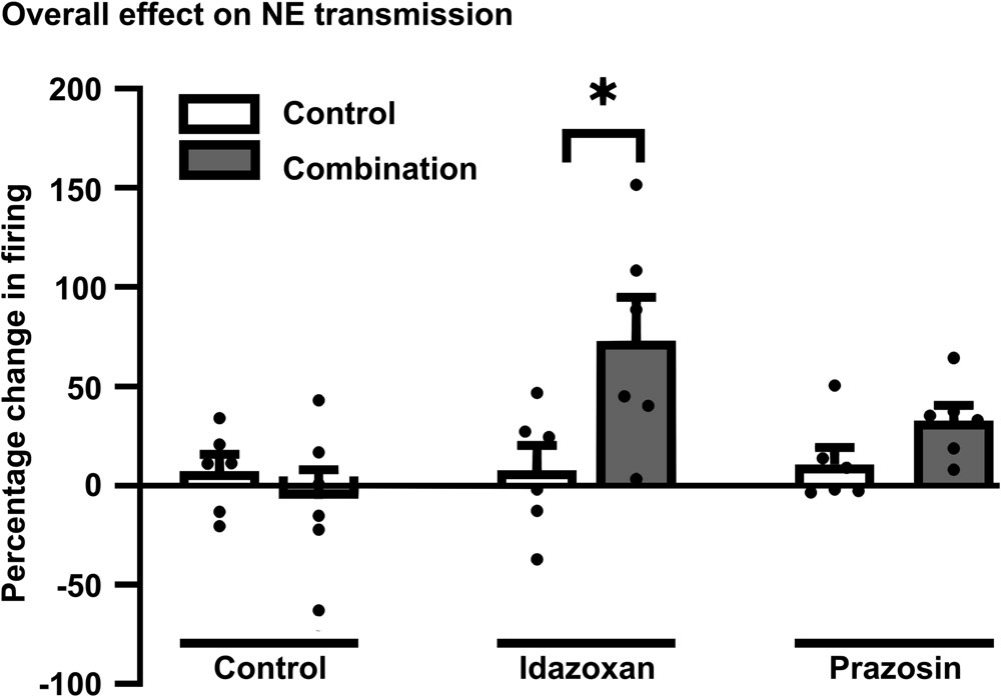
Effect of 14-day administration of the combination of venlafaxine and brexpiprazole on the tonic activation of α_2_- and α_1_-adrenoreceptor on CA3 hippocampal pyramidal neurons. Histograms illustrating the percentage change in firing of pyramidal neurons following acute administration of the α_2_-adrenoreceptor antagonist idazoxan (1 mg/kg) and the α_1_-adrenoreceptor antagonist prazosin (0.1 mg/kg) in a 14-day administered vehicle and 14-day combination-administered rat.

For the tonic activation of α_-_adrenoceptors, two-way ANOVA indicated a significant main effect of treatment (Fig. 6; *F* [1,30] = 5, *p* = 0.03) and drugs (*F* [2,30] = 5, *p* < 0.02), but no interaction between group and drugs (*F* [2,30] = 4, *p* = 0.02). Posthoc analysis using Holm–Sidak method showed that there was a significant increase in the percentage change in firing activity of CA3 pyramidal neurons induced by idazoxan versus saline in combination compared to control.

## Discussion

Short-term administration of the combination significantly enhanced VTA DA neuron activity, which was attenuated by an AMPA receptor antagonist. Previously observed venlafaxine-induced dampening of NE and 5-HT neuron activity with a two-day regimen was not observed when brexpiprazole was added herein. Furthermore, sustained administration of this combination increased 5-HT and NE transmission as determined in a projection area namely the dorsal hippocampus. This demonstrated a net overall enhancement of these monoaminergic systems.

### DA system

In the current study, short-term administration of venlafaxine significantly reduced the activity of DA neurons in the VTA. This inhibition can be attributed to the increase in the inhibitory tone of NE and 5-HT following blockade of NET and 5-HTT, respectively. A similar suppression of firing was also observed with an increase of 5-HT and NE following a 2-day administration of the SSRI escitalopram and the NRI reboxetine, respectively [6, 39]. However, it is uncertain why this activity returned to baseline level after 14-day administration of venlafaxine, while 5-HT and NE levels are still elevated. Surprisingly, not only the mean firing of DA neurons normalized, but in addition, the number of spontaneously active neurons increased per electrode descent after long-term administration of venlafaxine. It is possible that the venlafaxine-induced increase in population activity took place indirectly through an action on the prefrontal cortex. This is strengthened by the fact that electrical stimulation of medial prefrontal cortex (mPFC) pyramidal neurons, especially in burst mode, yielded an enhancement in the number of spontaneously active DA neurons in the VTA [36]. In the case of venlafaxine, the increase in population activity that resulted potentially from excitation of pyramidal neurons in the cortex could not be due to blockade of 5-HTT because the 5-HTT blocker escitalopram did not result in an increase of mPFC pyramidal neurons firing [40]. However, it may have happened through inhibition of NET, since desipramine significantly increases firing activity of mPFC pyramidal neurons [41]. The latter increase could have also been due to blockade by brexpiprazole of the terminal α_2C_-adrenoceptor, although this needs to be determined.

In addition, when brexpiprazole was combined with venlafaxine, the number of spontaneously active DA neurons was still increased. This may have been due to its antagonism at D_2_R, which increases firing activity of the nucleus accumbens, leading to a gamma-aminobutyric acid (GABA) inhibition of activity of ventral pallidum, hence releasing DA neurons from inhibition. This then results in an increase in population activity of DA neurons [37, 42]. Nevertheless, a glutamate action from the mPFC on the population activity of DA neuron cannot be excluded [36]. Should this be the case, then blockade of the AMPA receptor should cancel it out. Indeed, administration of the AMPA receptor antagonist NBQX blocked the increase in the number of spontaneously active DA neurons induced with the combination. This blockade must have taken place in the VTA since AMPA has an excitatory influence of DA neurons [43, 44]. These results highlight the importance of the glutamate system, particularly AMPA receptors, in the antidepressant response to drugs with affinity exclusively for monoamine targets. Indeed, there are many interactions between these two systems. The SSRI fluoxetine increases phosphorylation of GluA1 AMPA receptor subunits, hence resulting in enhancement of AMPA receptor signaling [45, 46]. The increase in 5-HT release in the mPFC produced by the NMDA antagonist MK-801 is also blocked by NBQX [47, 48]. On the other hand, NBQX blocks the ketamine-induced increase in DA neurons population activity in rats [38]. Altogether, these lines of evidence suggests that increased AMPA receptor signaling might represent a common mechanism underlying antidepressant action. Indeed, an increase in AMPA throughput is suggested to underlie the antidepressant effect of ketamine [49].

The current data show that the venlafaxine-induced inhibition of DA neuronal firing after 2 days was rescued by the addition of brexpiprazole, which by itself has no effect on DA neurons [16]. Since brexpiprazole possesses its highest affinity for 5-HT_1A_R, it is possible that this normalization of DA firing occurred through an action on this receptor, as 5-HT_1A_R agonists do [50–54]. In addition, since brexpiprazole also has a high and moderate affinity, respectively, for α_2C_- and α_2A_-adrenoceptors (Ki = 0.6 nM and 15 nM) [55], it is possible that this normalization occurred through an action on this receptor, as it was shown that blockade of α_2_-adrenoceptors by idazoxan increased firing activity of DA neurons [56].

### 5-HT system

Previous data demonstrated that a 2-day regimen of venlafaxine decreased firing rates of 5-HT neurons due to activation of the 5-HT_1A_ autoreceptor [12], while brexpiprazole significantly increased this rate [16]. Their combination as found in the current study, resulted in 5-HT neurons firing at baseline level. Hence, it is likely that the increasing effect of brexpiprazole on the firing activity of 5-HT neurons at 2 days counteracted the dampening effect of venlafaxine. This early normalization of firing is, however, unlikely due to desensitization of the 5-HT_1A_ autoreceptor because the 2-day administration of brexpiprazole did not affect sensitivity of this autoreceptor [16], although it is desensitized with venlafaxine after 14 days of administration [12]. This recovery could rather be mediated by an enhanced action on D_2_Rs located on 5-HT neurons [57].

In the hippocampus, our previous studies have shown that venlafaxine and brexpiprazole on their own significantly increase the tonic activation of postsynaptic 5-HT_1A_Rs [12, 16]. Although the present study showed an enhancement in the tonic activation of 5-HT_1A_Rs in the combination group, this did not seem to be dependent on the dose of WAY 100635. While the sensitivity of these receptors remained normal, this enhancement in tonic activation may constitute a direct functional evidence of a sustained enhancement of 5-HT neurotransmission. Although brexpiprazole is a 5-HT_1A_R agonist, this property does not underlie the increase shown in tonic activation both at 2 and 14 days of administration, because acute administration of brexpiprazole did not induce tonic activation of the 5-HT_1A_R [16]. A desensitization of the terminal 5-HT_1B_ autoreceptor could not account for this increase in 5-HT transmission. Indeed, despite this receptor being desensitized in the presence of venlafaxine [12], this effect was not present herein following combination with brexpiprazole, which by itself does not affect the sensitivity of this terminal autoreceptor [16]. Instead, the enhancement of 5-HT transmission could rather be the result of 5-HT reuptake inhibition by venlafaxine and a blockade by brexpiprazole of the α_2_-heteroreceptor on 5-HT terminals, as previously shown [58].

### NE system

It was previously reported that venlafaxine on its own significantly decreased firing activity of LC NE neurons [12], while brexpiprazole increased it after 2 and 14 days of administration [16]. In the current study, the combination of brexpiprazole with venlafaxine resulted in NE neurons firing at their basal level, demonstrating the efficacy of brexpiprazole to reverse a reduction that occurs with venlafaxine alone. It is unlikely that this normalization is due to blockade of α_2C_-adrenoceptors in the LC, as brexpiprazole neither blocked nor reversed the inhibitory effect of the non-selective α_2_-adrenergic agonist clonidine on NE neuronal firing activity. This indicated that cell body α_2-_autoreceptors are not of α_2C_-adrenoceptors subtype, but mostly of the α_2A_ subtype [16, 59]. Also, a change in the sensitivity of the somatodendritic α_2_-adrenergic autoreceptors could not account for normalization because neither venlafaxine nor brexpiprazole induced a desensitization of this autoreceptor [16, 17, 60]. Since brexpiprazole blocks 5-HT_2A_R [16], it is probable that it antagonized those on GABA neurons controlling the firing of LC NE neurons, hence preventing the dampening effect of venlafaxine on NE neuronal activity. Furthermore, since brexpiprazole possesses a very high affinity for this receptor, it is likely that the agonistic action of brexpiprazole on 5-HT_1A_Rs may have increased NE neuronal activity, as 5-HT_1A_R agonists such as 8-OH-DPAT and gepirone do [17, 61].

Steady-state administration of high-dose venlafaxine has been shown to inhibit the transporter for NE [12]. In the presence of brexpiprazole, however, venlafaxine did not attenuate NE reuptake in the hippocampus, yet when tested again herein, venlafaxine administration alone for 2 days blocked the NET (Table 1B). It is conceivable that the unaltered function of NET by venlafaxine is due to the potential interaction of brexpiprazole, which have several receptorial targets, as was previously reported for 5-HTT [62, 63]. Nonetheless, an additive/potentiated effect of NET with α_2_-adrenoceptor blockade on NE levels was reported [64, 65]. Interestingly in the present study, sustained combination administration significantly increased the tonic activation of the postsynaptic α_2_-adrenergic receptor in the hippocampus, revealing an augmented net overall effect of NE transmission. This enhancement was not due to an increased sensitivity of postsynaptic α_2_-adrenergic receptors. Although the effect of venlafaxine on the terminal α_2_-adrenergic autoreceptor was not determined, it is possible that the increased net NE transmission resulted from the potent antagonistic activity of brexpiprazole for the α_2C_-adrenoceptor, since α_2C_-adrenergic antagonism is involved in increasing NE levels [66, 67]. This is further strengthened by data showing that a combination of venlafaxine with the blockade of α_2_-adrenoceptors by idazoxan increased NE levels in the hippocampus and prefrontal cortex [64, 68]. Altogether, these results show that addition of brexpiprazole to venlafaxine resulted in NE neurons firing at normal levels as early as 2 days.

In summary, this study showed that combination of venlafaxine and brexpiprazole induces an increase in population activity of DA neurons that is modulated by AMPARs. It also revealed that the venlafaxine-induced decrease of 5-HT and NE neurons firing is promptly normalized by combination with brexpiprazole. This results in an enhancement of the tonic activation of 5-HT_1A_Rs and α_2_-adrenoceptors in the hippocampus. Altogether, these results indicate that such a strategy could be a rapid antidepressant oral treatment.

## Data Availability

Data are shown in the figures, and material and raw data for the analysis will be provided upon request.
